# Effects on milk quantity and composition associated with extruded linseed supplementation to dairy cow diets

**DOI:** 10.1038/s41598-019-54193-z

**Published:** 2019-11-26

**Authors:** Juan Manuel Ariza, Thomas Meignan, Aurélien Madouasse, François Beaudeau, Nathalie Bareille

**Affiliations:** 1 BIOEPAR, INRA, Oniris, La Chantrerie, F-44307 Nantes, France; 2 Valorex, La Messayais, F-35210 Combourtillé, France

**Keywords:** Systems biology, Risk factors

## Abstract

Enhanced milk composition can improve human health. The composition of milk determines its nutritional and market value. Therefore, in almost all pricing schemes the economic benefits obtained from raw milk sales are influenced by the milk yield and composition. The objective of this retrospective study was to quantify the average effects of supplementing extruded linseed, rich in α-linolenic acid, to dairy cows on milk yield and milk fat and protein content under field conditions. The study included test day records performed on cows from 1294 dairy herds during the period from 2008 to 2015 that were supplied at least 4 times with extruded linseed deliveries. Exposure statuses were defined according to the time sequence and the amount of extruded linseed distributed in the herd. The unexposed population was composed of cows being in a herd period when extruded linseed was not offered. In a linear dose-response relationship, every 100 g increase in exposure to EL was associated with an increased milk yield from 0.11 to 0.14 kg/day, decreased milk fat from 0.06 to 0.13 g/kg and decreased milk protein from 0 to 0.02 g/kg, according to the cow parity. This study provides information on the associations between estimated intakes of EL and milk production and composition using a large database obtained from commercial dairy herds.

## Introduction

Milk and dairy products constitute an important part of human diet^1^. Concerning human health, there is an opportunity to improve the fatty acid (FA) intake of humans^2^ by increasing polyunsaturated fatty acids (PUFA) at the expense of saturated fatty acids (SFA) or by rebalancing the ratio n-6/n-3^3,4^ as milk and dairy products represent a major part of FA supply and especially SFA^5^. Extruded linseed (EL) is a source of fat of which 54% of total FA is α-linolenic acid (ALA, 18:3 n-3)^5^. Its incorporation into dairy cow diets could modify in a linear dose-response manner the milk FA profile^6^. There has been a growing use of EL in French farms in the last decade, primarily to modify the milk FA profile^7^, but also to improve the reproductive performance of cows^8^. Nevertheless, the productive benefits of EL supplementation are not clearly known^6^.

Feeding dairy cows with high-fat feeds could enhance their energy balance status by increasing the dietary energy density without increasing the dietary starch content. Consequently, supplemental fat in the diet could improve milk yield (MY) in particular during early lactation when energy supply does not meet the requirements for maintenance and production^9^. However, selecting the proper dietary fat content is an important management decision that may affect the success of this supplementation^10^. Considering amounts of fat maybe even more relevant when supplementing PUFA because of the formation of ruminal biohydrogenation intermediates that exert anti-lipogenic effects leading to a decrease in milk fat content (MFC)^11,12^. Recently, a meta-analysis of experimental trials, revealed a mean increase effect on MY of +0.72 kg/day when supplementing EL inside a practical range (i.e., <600 g/cow/day) whereas no effect was observed when considering high-fat diets^6^.

Likewise, MFC decreased in dairy cows supplemented with EL at high inclusion level or when associated with high corn silage-based diets^6^.

Whilst it is recognized that supplementing EL in field conditions is restricted because of its high cost^13^, it is unclear what impact might have this strategy on milk quantity and composition. Furthermore, only a few experiments have evaluated the effects associated with supplementing EL at low levels such as those used in commercial farms and explored variation inside this practical range. Consequently, there is a considerable need to elucidate how EL supplementation under field conditions may enhance the sustainability of dairying.

This observational study aimed to quantify the average effects of supplementing EL to dairy cows on MY and milk composition (fat and protein) under field conditions. This was undertaken by assessing the EL exposure and evaluating the test day records of French dairy cattle from 2008 to 2015 using linear models.

## Methods

### General study design and available data

A retrospective observational study was conducted on data from French dairy herds enrolled in the official Milk Recording Scheme and wherein dairy cows were supplemented with EL between January 2008 and December 2015. Milk performance of cows at each test day (cow-TD, over 24 h) during periods of EL supplementation was compared to cow-TDs during periods of EL non-supplementation within the same herd. The EL exposure data were obtained from commercial companies delivering products containing EL during the study period. The TD information obtained from the official Milk Recording Scheme Lactation includes the date of TD, MY, MFC and milk protein content (MPC), calving date, parity and animal movement data.

### Estimation of exposure to extruded linseed and determination of exposure status

A cow daily exposure to EL for each delivery in each herd was calculated from the time-line of product delivery distribution, the quantity delivered, the products’ EL content, and the average number of lactating cows in the herd during the period of delivery distribution. The beginning of EL supplementation to dairy cows in each herd was considered to occur immediately the day after the date of feed arrival on the farm. The number of cows by herd and day was calculated based on movement data and test-day records. Doing that, we considered that all cows (i.e., whatever their lactation stage and MY) were supplemented with the same EL quantity within a herd. When a farmer received supplies of several feeds containing EL at the same time (i.e., on the same day), herd daily exposures from each delivery were added. In the end, a mean EL intake by cow by herd for each day of the study period was calculated.

For every cow-TD, an exposure status was determined according to the exposure of the herd at the moment of the TD. A cow-TD was considered exposed only if the cow was supplemented with EL continuously since the previous TD (or since its calving in the case of the first TD within a lactation). For each cow-TD, an average daily EL exposure during the interval between TD and previous TD (or TD and calving) was calculated by adding each daily exposure (from each day of this interval), estimated as described above, divided by the number of days of this interval. Based on the findings of a recent meta-analysis and on recommendations for EL use in practice, daily intakes higher than 1500 g/cow/day (0.13% of cow-TD) were considered plausible and related lactations were removed from the dataset^6^. The precision on the temporal sequence of exposure events was enhanced by excluding from the dataset all cow-TD records comprising the first TD after beginning EL supplementation and the first TD after stopping EL supplementation. In summary, the exposure variable accounted for both the dose and minimal time of exposure.

For each herd, all cow-TDs recorded in the Milk Recording Scheme during the study period were considered for the study. All the cow-TDs that were defined as not exposed constituted the reference population (unexposed category).

### Data selection

The initial study population consisted of 4979 French dairy herds having deliveries of EL during the study period. However, only 2250 herds were included due to a lack of herd identification number, and the lack of enrolment in the official Milk Recording Scheme. Furthermore, in order to ensure the heterogeneity of the EL exposure within and between herds, only herds receiving a minimum of four deliveries of feeds with EL during the study period were included in the dataset reducing the sample to 1836 herds. Likewise, the absence of fit between the delivery and the Milk Recording Scheme records, as well as missing data in deliveries reduced the sample to 1415 herds. Then, herds with unusual management (i.e., <10 calvings per year and with <15 or >65 percent of primiparous cows) were excluded reducing the sample to 1294 herds. Additionally, within herds, only data from Holstein cows were included because of the strong effect of breed on MY and milk contents. Only lactations that lasted at least 180 days, beginning after 1 January 2008 and before 6 July 2015 were included. In addition, cows and/or lactations with missing, implausible or unusual data were excluded: age at first calving <22 months or >40 months, calving to first TD <7 days, interval between TD <22 days or >73 days, milk contents equal to zero at TD. Finally, TDs occurring after 500 days in milk were excluded.

### Statistical models

The unit of measurement of the exposure was the herd-period of EL (non-) supplementation, while the milk performances were available at the cow-TD level. Therefore, the statistical modelling of the relationship between EL exposure and performance was done at the cow-TD level. The associations between MY, MFC and MPC and exposure to EL were modelled with linear mixed models. All the models had the following structure:

$${Y}_{tij}={X}_{tij}\beta +{\delta }_{j}+{\varepsilon }_{\begin{array}{c}tij\end{array}}$$

where *Y*_*tij*_ was the model outcome (MY, MFC or MPC) at TD *t* in cow *i* in herd *j*, *X*_*tij*_ was a matrix of covariates, *β* was a vector of associated coefficients, *ϑ*_*j*_ was a vector of herd random effects and *ε*_*tij*_ was a vector of residuals. The residuals were modelled as having a structure of autocorrelation of order 1 for cows between consecutive TD in the same lactation (see ref.^17^ for a more complete description of this model).

The matrix *X*_*tij*_ included EL (no)-supplementation, which was the variable of interest, as well as other cow and environmental factors likely to influence milk performance^14,15^. These other factors were the age at first calving (for records of primiparous cows only), days in milk, gestation stage (8 categories), the month of TD (12 categories), year of TD (8 categories), geographic area (7 categories). Days in milk were modelled using linear splines with knots at 30, 65, 125, 245, 305 and 365 days in milk as suggested by Bohmanova *et al*.^16^. In order to account for the fact that lactation curve shapes differ between parities^15^ and for possible different effects of EL supplementation between parities, models were stratified by parity, i.e. the model coefficients were estimated separately for cows of parity 1, 2, 3, 4 and greater than 4.

The statistical unit was, therefore, the cow-TD. The associations of interest were between model outcomes and EL (non-)supplementation. Model outcomes, as well as the other covariates included, were available at the cow-TD level, while EL supplementation was estimated at the herd TD level and considered to be the same for all cows recorded on the same TD. Finally, the linearity of the association was checked by first including exposure to EL as a categorical variable with 4 levels of exposure (“1–50”, “51–300”, “301–600” and “601–1500” (g/cow/day), Supplementary Table S6). Having checked that the relation was linear, EL exposure was included as a continuous variable in the models. Including EL exposure as a continuous variable, as opposed to categorical, resulted in decreases in model AIC ranging from 62 to 4294.

All statistical analyses were performed in R (version 3.3.2)^17^ using the nlme^18^ package and visualized using the plotly^19^ package. P-values ≤ 0.05 were considered significant.

## Results

### Distribution of data and unadjusted milk performance

The final sample was composed of 1294 herds, 196426 cows, and 404161 lactations, and 3847319 TDs (Table 1). Almost half of the TDs selected were unexposed. Nearly 80% of the exposed TDs received concentration ranging between 51 and 600 g/cow/day of EL. The mean daily EL intake in the exposed population was 311 (±234.20) g/cow/day. Means of MY, MFC and MPC were respectively 28.03 (±8.12) kg/day, 40.11 (±6.84) g/kg and 32.55 (±3.73) g/kg in the unexposed population, and respectively 29.19 (±8.33) kg/day, 39.40 (±6.67) g/kg and 32.40 (±3.62) g/kg in the whole exposed population. The number of TDs decreased with increasing parity with more than 36% of TDs related to primiparous cows (Table 2). The mean MY increased linearly by parity up to the 4 parity and the MFC increased from parity 1 to parity 5 + (Table 1), whereas the MPC reached a maximum concentration at parity 2 and then decreased.

**Table 1:** Unadjusted means of milk yield (MY).

		EL Exposure status^a^				
		Unexposed	Q1	Q2	Q3	Q4
Herds		1260	812	992	936	642
Cows		151204	63258	80376	79051	54601
Lactations		272841	94712	120677	124121	89336
Test day records		2134520	429431	427657	428186	427525
EL^2^ (g/cow/day)	Mean	0	67.66	194.21	343.91	641.52
	SD	0.0	37.95	36.78	52.30	175.35
MY (kg/day)	Parity 1	25.55	25.09	26.42	26.96	27.55
	Parity 2	28.98	28.64	30.15	30.70	31.40
	Parity 3	29.93	29.65	31.24	31.80	32.58
	Parity 4	29.89	29.66	31.22	31.81	32.62
	Parity 5+	28.22	27.70	29.59	29.84	30.64
MFC (g/kg)	Parity 1	39.49	39.56	39.17	38.81	38.50
	Parity 2	40.22	40.17	39.58	39.21	38.79
	Parity 3	40.51	40.44	39.77	39.37	38.98
	Parity 4	40.75	40.56	40.07	39.56	39.06
	Parity 5+	40.72	40.90	40.32	39.93	39.77
MPC (g/kg)	Parity 1	32.35	32.38	32.30	32.23	32.13
	Parity 2	32.87	32.92	32.73	32.64	32.48
	Parity 3	32.64	32.73	32.50	32.37	32.21
	Parity 4	32.50	32.55	32.35	32.20	32.04
	Parity 5+	32.11	32.25	31.95	31.80	31.71

Milk fat content (MFC) and milk protein content (MPC) per test day (TD) according to extruded linseed (EL) exposure status and stratification factors in 1294 French Holstein dairy herds during the study period 2008–2015 (n = 3847319 cow test-day records)^1^. Exposure status was gathered according to the quartiles of average daily intake of EL per cow per day during the interval between two TD or between calving and first TD.

**Table 2:** Model results for the effects on milk quantity and composition for every 100 g increase in exposure to EL.

Performance studied	Parity																			
	1 (n = 1373175)				2 (n = 1060457)				3 (n = 695611)				4 (n = 398052)				5 + (n = 320024)			
	Estimate	SE	*P*^a^	Φ^b^	Estimate	SE	*P*	Φ	Estimate	SE	*P*	Φ	Estimate	SE	*P*	Φ	Estimate	SE	*P*	Φ
MY (kg/day)	0.113	0.003	<0.001	0.68	0 .129	0.004	<0.001	0.70	0.136	0.005	<0.001	0.68	0.147	0.007	<0.001	0.67	0.121	0.008	<0.001	0.67
MFC (g/kg)	−0.063	0.003	<0.001	0.60	−0.099	0.004	<0.001	0.58	−0.101	0.006	<0.001	0.55	−0.133	0.008	<0.001	0.53	−0.108	0.009	<0.001	0.51
MPC (g/kg)	−0.006	0.001	<0.001	0.72	−0.012	0.001	<0.001	0.75	−0.019	0.002	<0.001	0.74	−0.023	0.003	<0.001	0.72	−0.022	0.003	<0.001	0.72

Exposure doses were calculated according to the average daily intake of EL per cow per day during the interval between two TD or between calving and first TD^a^. *P*-value^b^. Estimation of the parameter Φ of the first-order autoregressive correlation structure.

### Dose-dependent increase of milk quantity and composition associated with exposure to extruded linseed

**Figure 1:**
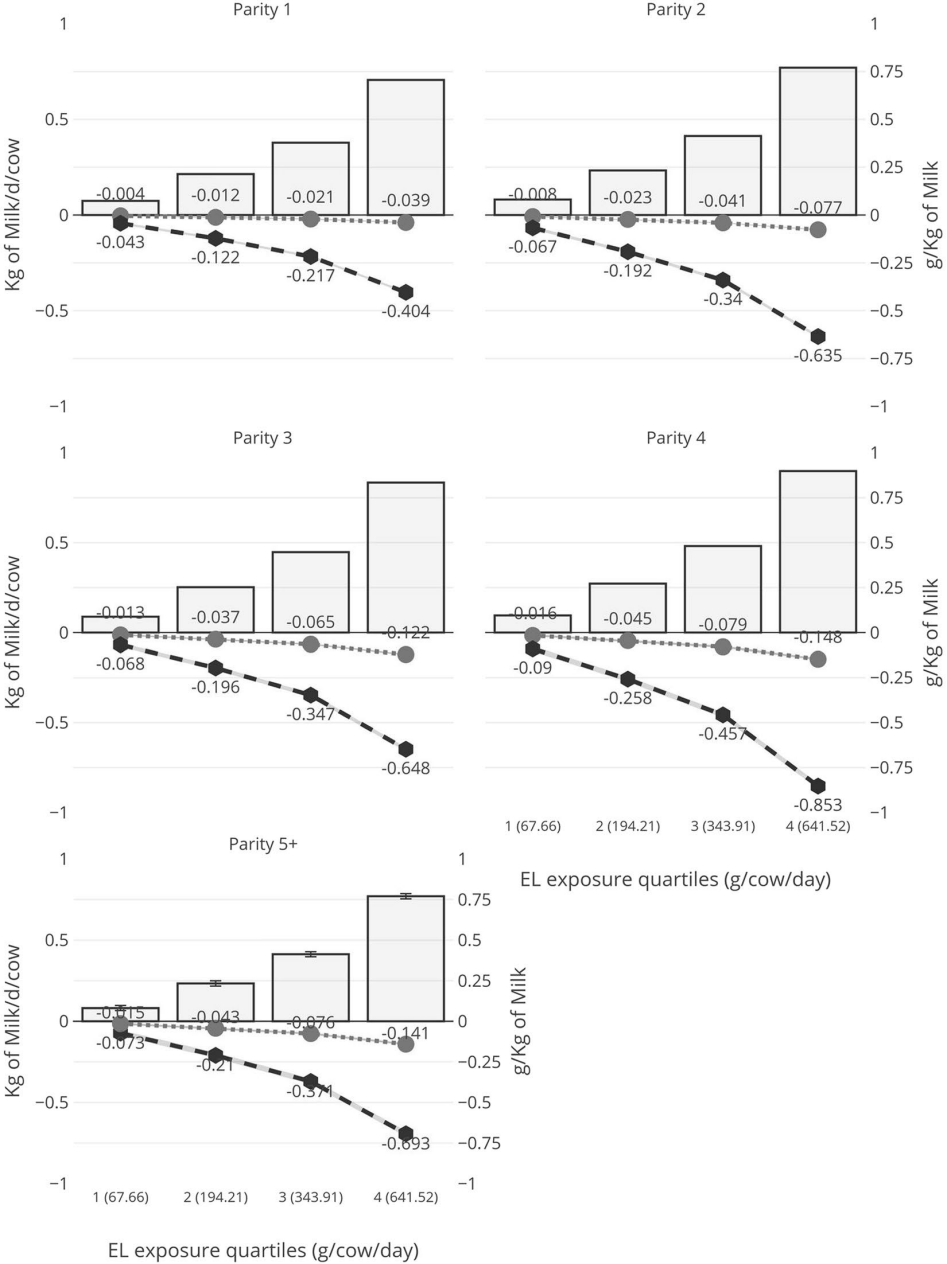
Model results for the association between extruded linseed exposure status and milk yield and composition according to each parity in 1294 French Holstein dairy herds during the study period 2008–2015 (n = 3847319 cow test-day records). Exposure status was gathered according to the quartiles of average daily intake of EL per cow per day during the interval between two TD or between calving and first TD. Bars represent the milk yield (Kg/day/cow), black hexagons line the milk fat content (g/Kg) and grey circles line the milk protein content (g/Kg).

For every 100 g increase in exposure to EL, MY increased by 0.113, 0.129, 0.137, 0.147 and 0.121 kg/day for cows of parity 1, 2, 3, 4 and greater than 4 respectively (Table 2). Across quartiles of daily EL exposure, the MY increased on average from 0.29 to 2.76% for parity 1, from 0.28 to 2.65% for parity 2, from 0.29 to 2.78% for parity 3, from 0.31 to 3.00% for parity 4 and from 0.28 to 2.72% for parity 5 + (Table 2 and Fig. 1). For every 100 g increase in exposure to EL, MFC decreased by −0.063, −0.099, −0.101, −0.133 and −0.108 kg/day for cows of parity 1, 2, 3, 4 and greater than 4 respectively (Table 2). Across quartiles of daily EL exposure, the MFC decreased on average from 0.10 to 1.02% for parity 1, from 0.16 to 1.59% for parity 2, from 0.16 to 1.57% for parity 3, from 0.22 to 2.09% for parity 4 and from 0.17 to 1.70% for parity 5 + (Table 2 and Fig. 1). For every 100 g increase in exposure to EL, MPC decreased by −0.006, −0.012, −0.019, −0.023 and −0.022 kg/day for cows of parity 1, 2, 3, 4 and greater than 4 respectively (Table 2). Across quartiles of daily EL exposure, the MPC decreased on average from 0.01 to 0.12% for parity 1, from 0.02 to 0.23% for parity 2, from 0.03 to 0.37% for parity 3, from 0.04 to 0.45% for parity 4 and from 0.04 to 0.43% for parity 5 + (Table 2 and Fig. 1).

### Adjustment variables associated with milk performance

For all parities, the adjustment variables included in the models were associated with MY, MFC and MPC. The complete results of the models are presented separately in Supplementary Tables S1, S2, S3, S4, and S5. The coefficients associated with the linear spline functions of days in milk were significant and the predicted curves were consistent with current knowledge on milk production. Otherwise, MY trended to decrease and MFC and MPC to increase as days in gestation increased. Milk yield trended to increase during months of spring compared to months of autumn and winter while MFC and MPC trended to decrease during months of spring and summer compared to months of winter. Across parities, MY minimal and maximal estimates were recorded at the years 2008 and 2015, respectively and except for parity 1. Similarly, regarding the geographic areas across parities, MY minimal and maximal estimates were recorded on “Field crop areas” and “Mountains wetlands in the Massif Central”, respectively. Finally, although effects on MFC and MPC were associated with the year and the geographic areas studied, no trends were observed between or within parities for these variables.

## Discussion

This study based on a large dataset composed of 404161 lactations recorded on 1294 herds and was to our knowledge the first large field-based epidemiological study exploring the association between nutrition (i.e., exposure to a feed) and dairy cow milk performance. The high statistical power resulting from our large sample size allowed us to demonstrate that supplementing EL in commercial farms could represent an economically viable strategy for enhancing dairy cow performance. Indeed, exposure to EL was associated linearly with an increase in MY, a decrease in MFC and, to a lesser extent in MPC. In France, private milk traders fix the price of raw milk adjusting for imbalances in the content of fat (38 g/L) and protein (32 g/L). Therefore, farmers supplementing EL may be expected to compensate for the decrease of MFC by the benefits of an increased MY.

Because of the observational nature of this study, the differences in milk production and constituents associated with EL supplementation cannot be attributed to the supplementation with certainty. There could be unmeasured factors associated with EL supplementation that resulted in changes in milk production. Additionally, this study suffers some limitations. First, the unexposed population was composed of cow-TDs recorded in herds that have been supplemented with EL, but during periods of EL non-supplementation, in order to reduce the potential for confounding from management practices. However, management alters over time and that confounding will exist in these data. Another option to avoid this bias could have consisted of selecting cow-TDs from herds that were never used EL-based feeds as the unexposed population. However, it would have presented many difficulties such as finding relevant matching criteria to study MY. Second, our approach implicitly assumes that the EL supplementation was, while purposive adequately controlled by periods when the herd does not use the EL. This assumption may be wrong, especially when the farmer considered not necessary or not cost-effective the EL supplementation. However, in France, EL is mainly considered as a local protein source for dairy cows, and when it is introduced in a diet, it replaces imported feeds such as soybean meal, in order to obtain iso-proteic diets. Therefore, EL being slightly more energetic than other protein sources, we could consider that the estimations provided might also include the general improvement of the diets containing EL. Third, we hypothesized also that all lactating cows within a herd were supplemented with the same quantity of EL whatever their days in milk. The true intake of EL for each cow could then have been under or overestimated since, in practice, cows in late lactation are less likely to be fed as much EL than cows in early and mid-lactation. However, we observed, within all the studied parities, associations between EL exposure and milk performance in a dose-dependent manner, suggesting that these methodological limits did not affect the consistency of the reported estimations, which were captured appropriately considering the mean herd exposure.

As an alternative to this herd approach, a prospective cohort study with a specific design to record diet composition for each group of cows could have increased the precision on EL exposure to reduce any intake-related bias. However, there is still a measurement error between the distribution of diet and true intakes by each cow due to cow variability in feed intake and the competition for access to feeds. Another option could have consisted of using biomarkers of specific intake which has a growing interest in human nutritional epidemiological studies^20,21^. The milk ALA content would be the target biomarker in the present study. Indeed, mid-infrared spectroscopy is available in routine and at an affordable price to estimate the milk FA profile. However, milk ALA content i) is still not well estimated by mid-infrared spectroscopy^22,23^ and ii) is closely dependent on ruminal biohydrogenation and modulated by other dietary components as grass or alfalfa^24^. Whatever the option is chosen to increase the reliability of EL exposure, such cohort studies would be difficult to carry out because of their cost and lack of practical feasibility.

Supplementing EL to dairy cows at a low level (first quartile 67.66 g of EL/cow/day) was associated with a slight impact on the dairy cow performance in all parities, as expected. Nevertheless, EL supplementation to dairy cows at the average quantity reported in this study 311 (±234.20) g/cow/day was associated with an increase in MY from 0.34 to 0.43 kg/day/cow. Supplementing EL at these moderate quantities may have increased energy density in the diet without negative effects such as lowering dry matter intake or reducing fiber digestibility in comparison with feeding high-unsaturated fat diets^10,25^. However, the magnitude of MY increase inside this range of EL supplementation was higher than previously reported. Indeed, in a recent meta-analysis of experimental trials, an average increase of MY of 0.70 kg/day was estimated by compiling 19 responses to EL intake with a mean intake of 715 g/cow/day^6^. A possible explanation for our finding is that diets in the field are less properly balanced compared to control diets used in experimental trials. Consequently, supplementing EL under field conditions may improve the beneficial effect on MY observed under experimental conditions.

The decrease in MFC associated with EL supplementation (range: 0.04 to 0.85 g/kg (first quartile parity1 and fourth quartile parity 4, respectively) was expected as EL contains PUFA. Indeed, adding PUFA to the dairy cow diet led to a decrease in milk fat precursors and to the formation of biohydrogenation intermediates inhibiting mammary milk fat synthesis^26,27^. Furthermore, this result was consistent with the dose-decrease of 0.30 g/kg by 250 g of EL estimated in a previous meta-analysis^6^. However, the nature of the forage in the diet could not be studied while this factor plays a major role in modulating milk fat responses in interaction with fat supplement^28,29^. For example, EL supplementation in high corn silage-based diet decreased MFC by 2.8 g/kg compared to EL supplementation in low corn silage-based diet^6^. Otherwise, according to a recent study, milk protein synthesis was not affected by supplementing any source of fat, including oilseeds^30^. Therefore, the slight decrease in MPC recorded in this study could result from a dilutive effect linked to the increased milk production of the exposed cows.

This study estimated the average daily intake of EL in the field of 311 g/cow/day. The cost of EL, as well as well-known negative effects of feeding high-fat diets, may restrict the quantity of EL supplemented in a commercial dairy herd diet. To our knowledge, very few experimental trials investigated the effects of such a low intake of EL on milk performance. The average estimated daily intake in our study represent 25% of experimental trials doses (1180 g/cow/day)^6^. High quantities of EL were supplemented in these experimental trials because they were mainly designed to study the evolution of milk FA profile after EL supplementation. The effect of EL on milk performance was seldom discussed in these trials. Finally, the present study was carried out on a large dataset designed to detect even small effects of a practical range of EL supplementation on milk performance if they exist. Indeed, we detected significant effects on milk performance of EL supplementation in low levels to dairy cows.

In conclusion, under field conditions, supplementing EL to dairy cows was associated with a linear increase in MY and a decrease in milk fat content. We clearly demonstrated that large scale epidemiological studies in commercial herds are complementary to experimental trials in order to quantify the associations between cow nutrition and cow performance, despite the lack of precise data on cow diets. To our knowledge, this is the first time an association between cow nutrition and milk performance was assessed in this way. Further studies under field conditions exploring the association between EL exposure and cow health and performance are needed to evaluate how dairy farmers willing to improve human health may benefit from this feeding practice.

## Supplementary information

Supplementary material

## Data Availability

The data that support the findings of this study are available, on reasonable request, from the corresponding author.
