# Supplementary material for: Effects on milk quantity and composition associated with extruded linseed supplementation to dairy cow diets
Source: Sci Rep. 2019 Nov 26;9:17563. doi: 10.1038/s41598-019-54193-z (PMC6879583; doi:10.1038/s41598-019-54193-z)
Supplement: Supplementary file 1 — Supplementary material [file 41598_2019_54193_MOESM1_ESM.docx]

**Supplementary material:**

Effects on milk quantity and composition associated with extruded linseed supplementation to dairy cow diets

Juan Manuel Ariza 1, Thomas Meignan 1,2, Aurélien Madouasse 1, François Beaudeau 1, and Nathalie Bareille 1*

1 BIOEPAR, INRA, Oniris, La Chantrerie, F-44307, Nantes, France

2 Valorex, La Messayais, F-35210 Combourtillé, France

*Corresponding author:

Email: juan-manuel.ariza@oniris-nantes.fr

**Supplementary Table S1.** Effect of adjustment variables on milk yield (MY)- milk fat content (MFC) and milk protein content (MPC) of 1 373 175 test-day records (TD) from 143 551 cows in first parity in 1 285 French Holstein dairy herds during the study period 2008-2015.

| **Variable** | **Parity 1** | | | | | | | | | | | |
| --- | --- | --- | --- | --- | --- | --- | --- | --- | --- | --- | --- | --- |
|  | **MY (kg/d)** | | | **MFC (g/kg)** | | | | **MPC (g/kg)** | | | | **Number of TD** |
|  | **Estimate** | **SE** | ***P^1^*** | **Estimate** | | **SE** | ***P^1^*** | **Estimate** | | **SE** | ***P^1^*** |  |
| **Intercept** | 18,472 | 0,133 | 0,000 | | 44,361 | 0,100 | 0,000 | | 32,794 | 0,050 | 0,000 |  |
| **Days in milk** | | | | | | | | | | | | |
| 0-30 | 4,497 | 0,024 | 0,000 | -8,052 | | 0,032 | 0,000 | -4,262 | | 0,012 | 0,000 | 93091 |
| 30-65 | 4,663 | 0,021 | 0,000 | -9,231 | | 0,028 | 0,000 | -3,277 | | 0,011 | 0,000 | 135885 |
| 65-125 | 3,009 | 0,024 | 0,000 | -7,868 | | 0,032 | 0,000 | -1,426 | | 0,012 | 0,000 | 237815 |
| 125-245 | 0,850 | 0,028 | 0,000 | -5,275 | | 0,036 | 0,000 | 0,638 | | 0,015 | 0,000 | 467386 |
| 245-305 | -0,591 | 0,031 | 0,000 | -3,823 | | 0,039 | 0,000 | 1,693 | | 0,016 | 0,000 | 204027 |
| 305-365 | -1,901 | 0,034 | 0,000 | -2,483 | | 0,043 | 0,000 | 2,939 | | 0,018 | 0,000 | 125340 |
| >365 | -4,275 | 0,046 | 0,000 | -0,916 | | 0,058 | 0,000 | 5,175 | | 0,025 | 0,000 | 109631 |
| **Gestation days** | | | | | | | | | | | | |
| No pregnancy | Ref | | | Ref | | | | Ref | | | | 465743 |
| (1-50] | -0,076 | 0,011 | 0,000 | 0,033 | | 0,015 | 0,030 | 0,054 | | 0,006 | 0,000 | 181584 |
| (50-100] | -0,321 | 0,016 | 0,000 | -0,214 | | 0,020 | 0,000 | 0,093 | | 0,008 | 0,000 | 175675 |
| (100-150] | -0,549 | 0,019 | 0,000 | 0,095 | | 0,024 | 0,000 | 0,000 | | 0,010 | 0,985 | 166983 |
| (150-200] | -1,065 | 0,022 | 0,000 | 0,598 | | 0,027 | 0,000 | 0,146 | | 0,012 | 0,000 | 154826 |
| (200-250] | -2,431 | 0,025 | 0,000 | 1,546 | | 0,031 | 0,000 | 0,472 | | 0,013 | 0,000 | 122097 |
| (>250] | -3,830 | 0,037 | 0,000 | 2,332 | | 0,048 | 0,000 | 0,962 | | 0,019 | 0,000 | 15905 |
| Not Determined | -2,381 | 0,036 | 0,000 | 0,824 | | 0,041 | 0,000 | 0,273 | | 0,020 | 0,000 | 90362 |
| **Month of TD** | | | | | | | | | | | | |
| January | 0,513 | 0,012 | 0,000 | -0,482 | | 0,016 | 0,000 | -0,421 | | 0,006 | 0,000 | 128882 |
| February | 0,745 | 0,014 | 0,000 | -0,613 | | 0,019 | 0,000 | -0,514 | | 0,007 | 0,000 | 119794 |
| March | 1,125 | 0,015 | 0,000 | -1,123 | | 0,020 | 0,000 | -0,767 | | 0,008 | 0,000 | 127829 |
| April | 1,303 | 0,016 | 0,000 | -1,992 | | 0,021 | 0,000 | -1,044 | | 0,008 | 0,000 | 120891 |
| May | 0,904 | 0,016 | 0,000 | -2,770 | | 0,021 | 0,000 | -1,530 | | 0,008 | 0,000 | 120102 |
| June | 0,415 | 0,016 | 0,000 | -3,228 | | 0,021 | 0,000 | -1,896 | | 0,008 | 0,000 | 120525 |
| July | 0,002 | 0,017 | 0,920 | -3,362 | | 0,022 | 0,000 | -2,087 | | 0,009 | 0,000 | 86703 |
| August | -0,299 | 0,018 | 0,000 | -2,668 | | 0,024 | 0,000 | -1,504 | | 0,009 | 0,000 | 63542 |
| September | -0,412 | 0,015 | 0,000 | -1,910 | | 0,020 | 0,000 | -0,839 | | 0,008 | 0,000 | 117888 |
| October | -0,467 | 0,013 | 0,000 | -0,999 | | 0,018 | 0,000 | -0,320 | | 0,007 | 0,000 | 124763 |
| November | -0,427 | 0,011 | 0,000 | -0,302 | | 0,015 | 0,000 | -0,026 | | 0,006 | 0,000 | 123799 |
| December | Ref | | | Ref | | | | Ref | | | | 118457 |
| **Year of TD** | | | | | | | | | | | | |
| 2008 | -0,897 | 0,032 | 0,000 | 0,286 | | 0,038 | 0,000 | -0,162 | | 0,017 | 0,000 | 67519 |
| 2009 | -0,912 | 0,024 | 0,000 | 0,346 | | 0,029 | 0,000 | -0,068 | | 0,013 | 0,000 | 171367 |
| 2010 | -0,588 | 0,023 | 0,000 | 0,568 | | 0,028 | 0,000 | 0,371 | | 0,012 | 0,000 | 176093 |
| 2011 | 0,031 | 0,020 | 0,115 | 0,052 | | 0,025 | 0,036 | -0,026 | | 0,011 | 0,013 | 193556 |
| 2012 | Ref | | | Ref | | | | Ref | | | | 192395 |
| 2013 | -0,474 | 0,020 | 0,000 | 0,121 | | 0,025 | 0,000 | 0,008 | | 0,011 | 0,434 | 198500 |
| 2014 | -0,193 | 0,022 | 0,000 | -0,053 | | 0,027 | 0,047 | -0,092 | | 0,012 | 0,000 | 204697 |
| 2015 | -0,035 | 0,025 | 0,161 | 0,383 | | 0,029 | 0,000 | 0,054 | | 0,014 | 0,000 | 169048 |
| **Geographic area** | | | | | | | | | | | | |
| Intensive areas in western France | Ref | | | Ref | | | | Ref | | | | 676049 |
| Field crops areas | 0,199 | 0,337 | 0,555 | -1,070 | | 0,163 | 0,000 | -0,333 | | 0,081 | 0,000 | 79272 |
| Grassland areas in northern Massif Central | -0,518 | 0,378 | 0,170 | -0,685 | | 0,184 | 0,000 | -0,496 | | 0,091 | 0,000 | 50416 |
| Grassland areas in northwestern France | -0,875 | 0,221 | 0,000 | -0,731 | | 0,108 | 0,000 | -0,323 | | 0,053 | 0,000 | 203450 |
| Intensive piedmont areas | -0,665 | 0,382 | 0,082 | -0,380 | | 0,188 | 0,043 | -0,377 | | 0,093 | 0,000 | 45121 |
| Mountains wetlands in Massif Central | -1,048 | 0,503 | 0,037 | -0,693 | | 0,243 | 0,005 | -0,391 | | 0,121 | 0,001 | 26446 |
| Mixed crop-livestock systems in Parisian Basin | 0,072 | 0,200 | 0,719 | -0,881 | | 0,096 | 0,000 | -0,249 | | 0,048 | 0,000 | 292421 |
| **Age at first calving** | 0,209 | 0,002 | 0,000 | 0,087 | | 0,003 | 0,000 | 0,028 | | 0,001 | 0,000 | 1373175 |
| ^1^ *P-*value for comparison with TD in the reference category | | | | | | | | | | | | |

**Supplementary Table S2.** Effect of adjustment variables on milk yield (MY). milk fat content (MFC) and milk protein content (MPC) of 1 060 457 test-day records (TD) from 111 159 cows in second parity in 1 288 French Holstein dairy herds during the study period 2008-2015.

| **Variable** | **Parity 2** | | | | | | | | | |
| --- | --- | --- | --- | --- | --- | --- | --- | --- | --- | --- |
|  | **MY (kg/d)** | | | **MFC (g/kg)** | | | **MPC (g/kg)** | | | **Number of TD** |
|  | **Estimate** | **SE** | ***P^1^*** | **Estimate** | **SE** | ***P^1^*** | **Estimate** | **SE** | ***P^1^*** |  |
| **Intercept** | 32,608 | 0,127 | 0,000 | 47,223 | 0,073 | 0,000 | 35,096 | 0,032 | 0,000 |  |
| **Days in milk** | | | | | | | | | | |
| 0-30 | 5,048 | 0,032 | 0,000 | -7,671 | 0,040 | 0,000 | -5,244 | 0,014 | 0,000 | 72208 |
| 30-65 | 3,571 | 0,028 | 0,000 | -8,910 | 0,034 | 0,000 | -4,373 | 0,013 | 0,000 | 105539 |
| 65-125 | -0,107 | 0,032 | 0,000 | -7,417 | 0,038 | 0,000 | -2,577 | 0,015 | 0,000 | 184313 |
| 125-245 | -5,526 | 0,037 | 0,000 | -4,653 | 0,043 | 0,000 | -0,153 | 0,018 | 0,000 | 359308 |
| 245-305 | -8,533 | 0,041 | 0,000 | -3,070 | 0,047 | 0,000 | 1,377 | 0,020 | 0,000 | 155882 |
| 305-365 | -11,106 | 0,046 | 0,000 | -1,731 | 0,052 | 0,000 | 2,923 | 0,022 | 0,000 | 100105 |
| >365 | -15,013 | 0,064 | 0,000 | -0,478 | 0,070 | 0,000 | 5,090 | 0,031 | 0,000 | 83102 |
| **Gestation days** | | | | | | | | | | |
| No pregnancy | Ref | | | Ref | | | Ref | | | 368193 |
| (1-50] | -0,230 | 0,015 | 0,000 | 0,033 | 0,019 | 0,078 | 0,045 | 0,007 | 0,000 | 135565 |
| (50-100] | -0,447 | 0,021 | 0,000 | -0,199 | 0,025 | 0,000 | 0,014 | 0,010 | 0,148 | 129415 |
| (100-150] | -0,730 | 0,026 | 0,000 | 0,073 | 0,029 | 0,012 | -0,086 | 0,012 | 0,000 | 121244 |
| (150-200] | -1,627 | 0,030 | 0,000 | 0,671 | 0,033 | 0,000 | 0,296 | 0,014 | 0,000 | 109517 |
| (200-250] | -3,597 | 0,034 | 0,000 | 1,595 | 0,037 | 0,000 | 0,879 | 0,017 | 0,000 | 79826 |
| (>250] | -4,655 | 0,052 | 0,000 | 2,136 | 0,060 | 0,000 | 1,272 | 0,024 | 0,000 | 10930 |
| Not Determined | -2,285 | 0,041 | 0,000 | 0,523 | 0,040 | 0,000 | 0,125 | 0,021 | 0,000 | 105767 |
| **Month of TD** | | | | | | | | | | |
| January | 0,508 | 0,016 | 0,000 | -0,427 | 0,020 | 0,000 | -0,488 | 0,007 | 0,000 | 97986 |
| February | 0,841 | 0,019 | 0,000 | -0,662 | 0,023 | 0,000 | -0,667 | 0,009 | 0,000 | 91581 |
| March | 1,436 | 0,021 | 0,000 | -1,404 | 0,025 | 0,000 | -0,992 | 0,009 | 0,000 | 98894 |
| April | 1,864 | 0,022 | 0,000 | -2,612 | 0,026 | 0,000 | -1,249 | 0,010 | 0,000 | 94133 |
| May | 1,445 | 0,022 | 0,000 | -3,402 | 0,026 | 0,000 | -1,717 | 0,010 | 0,000 | 93336 |
| June | 0,900 | 0,022 | 0,000 | -3,727 | 0,026 | 0,000 | -2,122 | 0,010 | 0,000 | 94155 |
| July | 0,317 | 0,023 | 0,000 | -3,844 | 0,027 | 0,000 | -2,395 | 0,010 | 0,000 | 67658 |
| August | -0,028 | 0,024 | 0,233 | -3,058 | 0,029 | 0,000 | -1,764 | 0,011 | 0,000 | 49625 |
| September | -0,251 | 0,020 | 0,000 | -2,199 | 0,025 | 0,000 | -1,001 | 0,009 | 0,000 | 91783 |
| October | -0,451 | 0,018 | 0,000 | -1,175 | 0,022 | 0,000 | -0,394 | 0,008 | 0,000 | 96221 |
| November | -0,495 | 0,015 | 0,000 | -0,288 | 0,019 | 0,000 | -0,027 | 0,007 | 0,000 | 94578 |
| December | Ref | | | Ref | | | Ref | | | 90507 |
| **Year of TD** | | | | | | | | | | |
| 2008 | -1,259 | 0,043 | 0,000 | 0,554 | 0,045 | 0,000 | -0,170 | 0,022 | 0,000 | 55548 |
| 2009 | -1,106 | 0,033 | 0,000 | 0,474 | 0,034 | 0,000 | -0,084 | 0,017 | 0,000 | 140651 |
| 2010 | -0,786 | 0,032 | 0,000 | 0,781 | 0,033 | 0,000 | 0,402 | 0,015 | 0,000 | 134163 |
| 2011 | -0,010 | 0,028 | 0,725 | 0,145 | 0,031 | 0,000 | -0,035 | 0,013 | 0,008 | 136388 |
| 2012 | Ref | | | Ref | | | Ref | | | 147901 |
| 2013 | -0,357 | 0,027 | 0,000 | 0,147 | 0,030 | 0,000 | -0,049 | 0,013 | 0,000 | 150768 |
| 2014 | 0,066 | 0,030 | 0,030 | -0,297 | 0,032 | 0,000 | -0,271 | 0,015 | 0,000 | 157895 |
| 2015 | 0,417 | 0,034 | 0,000 | 0,090 | 0,035 | 0,010 | -0,202 | 0,017 | 0,000 | 137143 |
| **Geographic area** | | | | | | | | | | |
| Intensive areas in western France | Ref | | | Ref | | | Ref | | | 527079 |
| Field crops areas | 0,023 | 0,369 | 0,950 | -1,082 | 0,182 | 0,000 | -0,351 | 0,084 | 0,000 | 60386 |
| Grassland areas in northern Massif Central | -0,664 | 0,416 | 0,111 | -0,520 | 0,207 | 0,012 | -0,451 | 0,096 | 0,000 | 38830 |
| Grassland areas in northwestern France | -1,177 | 0,242 | 0,000 | -0,687 | 0,121 | 0,000 | -0,201 | 0,056 | 0,000 | 153873 |
| Intensive piedmont areas | -1,304 | 0,420 | 0,002 | -0,110 | 0,209 | 0,599 | -0,192 | 0,097 | 0,049 | 37425 |
| Mountains wetlands in Massif Central | -1,860 | 0,558 | 0,001 | -0,477 | 0,275 | 0,083 | -0,255 | 0,127 | 0,045 | 20842 |
| Mixed crop-livestock systems in Parisian Basin | -0,184 | 0,220 | 0,403 | -0,712 | 0,108 | 0,000 | -0,254 | 0,050 | 0,000 | 222022 |
| ^1^ *P-*value for comparison with TD in the reference category | | | | | | | | | | |

**Supplementary Table S3.** Effect of adjustment variables on milk yield (MY)- milk fat content (MFC) and milk protein content (MPC) of 695 611 test-day records (TD) from 73 341 cows in third parity in 1 283 French Holstein dairy herds during the study period 2008-2015.

| **Variable** | **Parity 3** | | | | | | | | | |
| --- | --- | --- | --- | --- | --- | --- | --- | --- | --- | --- |
|  | **MY (kg/d)** | | | **MFC (g/kg)** | | | **MPC (g/kg)** | | | **Number of TD** |
|  | **Estimate** | **SE** | ***P^1^*** | **Estimate** | **SE** | ***P^1^*** | **Estimate** | **SE** | ***P^1^*** |  |
| **Intercept** | 34,426 | 0,131 | 0,000 | 48,887 | 0,081 | 0,000 | 35,001 | 0,033 | 0,000 |  |
| **Days in milk** | | | | | | | | | | |
| 0-30 | 5,511 | 0,041 | 0,000 | -8,211 | 0,052 | 0,000 | -5,458 | 0,018 | 0,000 | 48048 |
| 30-65 | 4,289 | 0,036 | 0,000 | -10,113 | 0,044 | 0,000 | -4,580 | 0,016 | 0,000 | 69758 |
| 65-125 | -0,206 | 0,041 | 0,000 | -8,615 | 0,049 | 0,000 | -2,628 | 0,018 | 0,000 | 122062 |
| 125-245 | -6,864 | 0,048 | 0,000 | -5,991 | 0,055 | 0,000 | -0,038 | 0,022 | 0,077 | 235769 |
| 245-305 | -10,256 | 0,053 | 0,000 | -4,534 | 0,059 | 0,000 | 1,519 | 0,024 | 0,000 | 101286 |
| 305-365 | -13,171 | 0,059 | 0,000 | -3,533 | 0,065 | 0,000 | 2,947 | 0,027 | 0,000 | 65401 |
| >365 | -17,217 | 0,083 | 0,000 | -2,830 | 0,090 | 0,000 | 4,681 | 0,038 | 0,000 | 53287 |
| **Gestation days** | | | | | | | | | | |
| No pregnancy | Ref | | | Ref | | | Ref | | | 231281 |
| (1-50] | -0,339 | 0,021 | 0,000 | 0,076 | 0,025 | 0,003 | 0,063 | 0,009 | 0,000 | 84441 |
| (50-100] | -0,615 | 0,028 | 0,000 | -0,072 | 0,032 | 0,026 | 0,022 | 0,013 | 0,079 | 79918 |
| (100-150] | -0,916 | 0,034 | 0,000 | 0,166 | 0,038 | 0,000 | -0,082 | 0,015 | 0,000 | 74009 |
| (150-200] | -1,880 | 0,040 | 0,000 | 0,738 | 0,043 | 0,000 | 0,348 | 0,018 | 0,000 | 66402 |
| (200-250] | -4,027 | 0,045 | 0,000 | 1,617 | 0,048 | 0,000 | 1,005 | 0,021 | 0,000 | 46844 |
| (>250] | -4,671 | 0,069 | 0,000 | 1,847 | 0,079 | 0,000 | 1,285 | 0,031 | 0,000 | 6712 |
| Not Determined | -2,329 | 0,045 | 0,000 | 0,400 | 0,042 | 0,000 | 0,052 | 0,022 | 0,017 | 106004 |
| **Month of TD** | | | | | | | | | | |
| January | 0,417 | 0,021 | 0,000 | -0,399 | 0,026 | 0,000 | -0,473 | 0,009 | 0,000 | 64302 |
| February | 0,737 | 0,025 | 0,000 | -0,662 | 0,031 | 0,000 | -0,709 | 0,011 | 0,000 | 60160 |
| March | 1,394 | 0,027 | 0,000 | -1,436 | 0,032 | 0,000 | -1,051 | 0,012 | 0,000 | 65200 |
| April | 1,892 | 0,028 | 0,000 | -2,688 | 0,033 | 0,000 | -1,277 | 0,012 | 0,000 | 61936 |
| May | 1,544 | 0,029 | 0,000 | -3,497 | 0,034 | 0,000 | -1,711 | 0,013 | 0,000 | 61728 |
| June | 1,035 | 0,029 | 0,000 | -3,822 | 0,034 | 0,000 | -2,165 | 0,013 | 0,000 | 61883 |
| July | 0,456 | 0,030 | 0,000 | -3,896 | 0,035 | 0,000 | -2,468 | 0,013 | 0,000 | 43987 |
| August | 0,078 | 0,031 | 0,013 | -3,074 | 0,038 | 0,000 | -1,810 | 0,014 | 0,000 | 32551 |
| September | -0,097 | 0,027 | 0,000 | -2,197 | 0,032 | 0,000 | -1,049 | 0,012 | 0,000 | 60287 |
| October | -0,283 | 0,024 | 0,000 | -1,234 | 0,029 | 0,000 | -0,413 | 0,010 | 0,000 | 62743 |
| November | -0,380 | 0,020 | 0,000 | -0,326 | 0,025 | 0,000 | -0,030 | 0,008 | 0,000 | 61923 |
| December | Ref | | | Ref | | | Ref | | | 58911 |
| **Year of TD** | | | | | | | | | | |
| 2008 | -1,291 | 0,056 | 0,000 | 0,609 | 0,056 | 0,000 | -0,111 | 0,026 | 0,000 | 39199 |
| 2009 | -1,114 | 0,043 | 0,000 | 0,410 | 0,043 | 0,000 | -0,105 | 0,020 | 0,000 | 93012 |
| 2010 | -0,877 | 0,041 | 0,000 | 0,789 | 0,042 | 0,000 | 0,398 | 0,019 | 0,000 | 93663 |
| 2011 | -0,058 | 0,037 | 0,116 | 0,337 | 0,039 | 0,000 | -0,004 | 0,017 | 0,818 | 92128 |
| 2012 | Ref | | | Ref | | | Ref | | | 89891 |
| 2013 | -0,261 | 0,036 | 0,000 | -0,004 | 0,039 | 0,918 | -0,083 | 0,016 | 0,000 | 97587 |
| 2014 | 0,375 | 0,040 | 0,000 | -0,568 | 0,041 | 0,000 | -0,354 | 0,019 | 0,000 | 103093 |
| 2015 | 0,793 | 0,045 | 0,000 | -0,346 | 0,045 | 0,000 | -0,348 | 0,021 | 0,000 | 87038 |
| **Geographic area** | | | | | | | | | | |
| Intensive areas in western France | Ref | | | Ref | | | Ref | | | 347656 |
| Field crops areas | 0,018 | 0,372 | 0,962 | -1,279 | 0,185 | 0,000 | -0,472 | 0,078 | 0,000 | 39042 |
| Grassland areas in northern Massif Central | -0,541 | 0,422 | 0,200 | -0,660 | 0,212 | 0,002 | -0,494 | 0,090 | 0,000 | 25735 |
| Grassland areas in northwestern France | -1,049 | 0,245 | 0,000 | -0,747 | 0,123 | 0,000 | -0,264 | 0,052 | 0,000 | 99620 |
| Intensive piedmont areas | -1,088 | 0,425 | 0,011 | -0,397 | 0,213 | 0,063 | -0,268 | 0,090 | 0,003 | 25944 |
| Mountains wetlands in Massif Central | -1,723 | 0,562 | 0,002 | -0,680 | 0,278 | 0,015 | -0,302 | 0,117 | 0,010 | 14667 |
| Mixed crop-livestock systems in Parisian Basin | -0,300 | 0,222 | 0,175 | -0,787 | 0,110 | 0,000 | -0,319 | 0,046 | 0,000 | 142947 |
| ^1^ *P-*value for comparison with TD in the reference category | | | | | | | | | | |

**Supplementary Table S4.** Effect of adjustment variables on milk yield (MY)- milk fat content (MFC) and milk protein content (MPC) of 398 052 test-day records (TD) from 42 138 cows in fourth parity in 1 277 French Holstein dairy herds during the study period 2008-2015.

| **Variable** | **Parity 4** | | | | | | | | | |
| --- | --- | --- | --- | --- | --- | --- | --- | --- | --- | --- |
|  | **MY (kg/d)** | | | **MFC (g/kg)** | | | **MPC (g/kg)** | | | **Number of TD** |
|  | **Estimate** | **SE** | ***P^1^*** | **Estimate** | **SE** | ***P^1^*** | **Estimate** | **SE** | ***P^1^*** |  |
| **Intercept** | 34,488 | 0,142 | 0,000 | 49,896 | 0,099 | 0,000 | 35,142 | 0,039 | 0,000 |  |
| **Days in milk** | | | | | | | | | | |
| 0-30 | 5,661 | 0,057 | 0,000 | -8,303 | 0,071 | 0,000 | -5,640 | 0,024 | 0,000 | 27291 |
| 30-65 | 4,745 | 0,049 | 0,000 | -10,371 | 0,061 | 0,000 | -4,742 | 0,021 | 0,000 | 40025 |
| 65-125 | -0,032 | 0,056 | 0,567 | -8,907 | 0,066 | 0,000 | -2,728 | 0,024 | 0,000 | 69919 |
| 125-245 | -7,136 | 0,064 | 0,000 | -6,430 | 0,073 | 0,000 | -0,179 | 0,028 | 0,000 | 135076 |
| 245-305 | -10,528 | 0,070 | 0,000 | -5,348 | 0,079 | 0,000 | 1,288 | 0,031 | 0,000 | 57150 |
| 305-365 | -13,491 | 0,078 | 0,000 | -4,403 | 0,086 | 0,000 | 2,615 | 0,035 | 0,000 | 37345 |
| >365 | -17,431 | 0,108 | 0,000 | -4,018 | 0,118 | 0,000 | 4,036 | 0,048 | 0,000 | 31246 |
| **Gestation days** | | | | | | | | | | |
| No pregnancy | Ref | | | Ref | | | Ref | | | 124228 |
| (1-50] | -0,378 | 0,029 | 0,000 | 0,030 | 0,035 | 0,389 | 0,037 | 0,012 | 0,003 | 44581 |
| (50-100] | -0,706 | 0,039 | 0,000 | -0,088 | 0,044 | 0,048 | -0,010 | 0,017 | 0,548 | 42280 |
| (100-150] | -1,057 | 0,047 | 0,000 | 0,178 | 0,051 | 0,001 | -0,097 | 0,021 | 0,000 | 39032 |
| (150-200] | -2,060 | 0,054 | 0,000 | 0,776 | 0,057 | 0,000 | 0,358 | 0,024 | 0,000 | 34420 |
| (200-250] | -4,268 | 0,061 | 0,000 | 1,643 | 0,065 | 0,000 | 1,053 | 0,027 | 0,000 | 23509 |
| (>250] | -4,780 | 0,094 | 0,000 | 1,796 | 0,108 | 0,000 | 1,332 | 0,041 | 0,000 | 3683 |
| Not Determined | -2,393 | 0,053 | 0,000 | 0,146 | 0,050 | 0,003 | -0,057 | 0,025 | 0,022 | 86319 |
| **Month of TD** | | | | | | | | | | |
| January | 0,397 | 0,029 | 0,000 | -0,483 | 0,035 | 0,000 | -0,510 | 0,012 | 0,000 | 36853 |
| February | 0,756 | 0,034 | 0,000 | -0,747 | 0,042 | 0,000 | -0,763 | 0,015 | 0,000 | 34367 |
| March | 1,430 | 0,037 | 0,000 | -1,537 | 0,043 | 0,000 | -1,101 | 0,016 | 0,000 | 37403 |
| April | 1,950 | 0,038 | 0,000 | -2,706 | 0,045 | 0,000 | -1,295 | 0,017 | 0,000 | 35515 |
| May | 1,648 | 0,039 | 0,000 | -3,488 | 0,046 | 0,000 | -1,699 | 0,017 | 0,000 | 35213 |
| June | 1,134 | 0,039 | 0,000 | -3,848 | 0,046 | 0,000 | -2,196 | 0,017 | 0,000 | 35336 |
| July | 0,509 | 0,041 | 0,000 | -3,921 | 0,048 | 0,000 | -2,498 | 0,017 | 0,000 | 25085 |
| August | 0,100 | 0,043 | 0,018 | -3,012 | 0,051 | 0,000 | -1,842 | 0,018 | 0,000 | 18517 |
| September | -0,041 | 0,036 | 0,260 | -2,190 | 0,043 | 0,000 | -1,054 | 0,016 | 0,000 | 34636 |
| October | -0,252 | 0,033 | 0,000 | -1,161 | 0,040 | 0,000 | -0,394 | 0,014 | 0,000 | 36116 |
| November | -0,365 | 0,027 | 0,000 | -0,318 | 0,034 | 0,000 | -0,018 | 0,011 | 0,120 | 35285 |
| December | Ref | | | Ref | | | Ref | | | 33726 |
| **Year of TD** | | | | | | | | | | |
| 2008 | -1,012 | 0,074 | 0,000 | 0,149 | 0,075 | 0,046 | -0,200 | 0,034 | 0,000 | 22852 |
| 2009 | -0,847 | 0,057 | 0,000 | 0,091 | 0,057 | 0,113 | -0,136 | 0,026 | 0,000 | 55527 |
| 2010 | -0,653 | 0,055 | 0,000 | 0,514 | 0,056 | 0,000 | 0,356 | 0,025 | 0,000 | 54365 |
| 2011 | 0,035 | 0,049 | 0,475 | 0,081 | 0,052 | 0,118 | -0,065 | 0,022 | 0,003 | 55410 |
| 2012 | Ref | | | Ref | | | Ref | | | 52038 |
| 2013 | -0,257 | 0,049 | 0,000 | -0,227 | 0,053 | 0,000 | -0,076 | 0,022 | 0,001 | 52458 |
| 2014 | 0,500 | 0,054 | 0,000 | -0,832 | 0,055 | 0,000 | -0,357 | 0,025 | 0,000 | 57559 |
| 2015 | 1,018 | 0,061 | 0,000 | -0,610 | 0,060 | 0,000 | -0,337 | 0,028 | 0,000 | 47843 |
| **Geographic area** | | | | | | | | | | |
| Intensive areas in western France | Ref | | | Ref | | | Ref | | | 200296 |
| Field crops areas | -0,016 | 0,383 | 0,966 | -1,169 | 0,197 | 0,000 | -0,512 | 0,083 | 0,000 | 22052 |
| Grassland areas in northern Massif Central | -0,589 | 0,436 | 0,177 | -0,600 | 0,227 | 0,008 | -0,539 | 0,096 | 0,000 | 14814 |
| Grassland areas in northwestern France | -0,944 | 0,254 | 0,000 | -0,805 | 0,131 | 0,000 | -0,321 | 0,055 | 0,000 | 57767 |
| Intensive piedmont areas | -1,297 | 0,430 | 0,003 | -0,569 | 0,224 | 0,011 | -0,418 | 0,095 | 0,000 | 16310 |
| Mountains wetlands in Massif Central | -1,582 | 0,575 | 0,006 | -0,908 | 0,294 | 0,002 | -0,413 | 0,123 | 0,001 | 9093 |
| Mixed crop-livestock systems in Parisian Basin | -0,388 | 0,228 | 0,090 | -0,906 | 0,117 | 0,000 | -0,425 | 0,049 | 0,000 | 77720 |
| ^1^ *P-*value for comparison with TD in the reference category | | | | | | | | | | |

**Supplementary Table S5.** Effect of adjustment variables on milk yield (MY)- milk fat content (MFC) and milk protein content (MPC) of 320 024 test-day records (TD) from 23714 cows in fifth or more parity in 1 268 French Holstein dairy herds during the study period 2008-2015.

| **Variable** | **Parity 5+** | | | | | | | | | |
| --- | --- | --- | --- | --- | --- | --- | --- | --- | --- | --- |
|  | **MY (kg/d)** | | | **MFC (g/kg)** | | | **MPC (g/kg)** | | | **Number of TD** |
|  | **Estimate** | **SE** | ***P^1^*** | **Estimate** | **SE** | ***P^1^*** | **Estimate** | **SE** | ***P^1^*** |  |
| **Intercept** | 33,735 | 0,152 | 0,000 | 49,922 | 0,113 | 0,000 | 34,954 | 0,045 | 0,000 |  |
| **Days in milk** | | | | | | | | | | |
| 0-30 | 5,703 | 0,062 | 0,000 | -7,882 | 0,081 | 0,000 | -5,578 | 0,027 | 0,000 | 22242 |
| 30-65 | 4,957 | 0,054 | 0,000 | -10,046 | 0,069 | 0,000 | -4,686 | 0,023 | 0,000 | 32336 |
| 65-125 | 0,073 | 0,061 | 0,232 | -8,533 | 0,075 | 0,000 | -2,647 | 0,026 | 0,000 | 56126 |
| 125-245 | -7,067 | 0,069 | 0,000 | -6,229 | 0,081 | 0,000 | -0,234 | 0,030 | 0,000 | 108470 |
| 245-305 | -10,406 | 0,075 | 0,000 | -5,401 | 0,087 | 0,000 | 1,142 | 0,033 | 0,000 | 44779 |
| 305-365 | -13,193 | 0,083 | 0,000 | -4,883 | 0,094 | 0,000 | 2,228 | 0,037 | 0,000 | 29742 |
| >365 | -16,992 | 0,115 | 0,000 | -4,682 | 0,128 | 0,000 | 3,296 | 0,051 | 0,000 | 26329 |
| **Gestation days** | | | | | | | | | | |
| No pregnancy | Ref | | | Ref | | | Ref | | | 90597 |
| (1-50] | -0,432 | 0,034 | 0,000 | 0,033 | 0,042 | 0,436 | 1,187 | 0,046 | 0,000 | 31267 |
| (50-100] | -0,849 | 0,045 | 0,000 | 0,030 | 0,052 | 0,559 | 0,043 | 0,014 | 0,003 | 29493 |
| (100-150] | -1,187 | 0,053 | 0,000 | 0,216 | 0,059 | 0,000 | -0,052 | 0,024 | 0,028 | 26856 |
| (150-200] | -2,288 | 0,060 | 0,000 | 0,793 | 0,066 | 0,000 | 0,481 | 0,027 | 0,000 | 23221 |
| (200-250] | -4,435 | 0,069 | 0,000 | 1,595 | 0,075 | 0,000 | 1,202 | 0,031 | 0,000 | 15360 |
| (>250] | -4,403 | 0,105 | 0,000 | 1,483 | 0,125 | 0,000 | 0,031 | 0,020 | 0,112 | 2850 |
| Not Determined | -2,441 | 0,054 | 0,000 | 0,086 | 0,050 | 0,089 | -0,044 | 0,025 | 0,078 | 100380 |
| **Month of TD** | | | | | | | | | | |
| January | 0,461 | 0,032 | 0,000 | -0,450 | 0,040 | 0,000 | -0,549 | 0,014 | 0,000 | 29693 |
| February | 0,844 | 0,038 | 0,000 | -0,725 | 0,047 | 0,000 | -0,840 | 0,016 | 0,000 | 27615 |
| March | 1,481 | 0,040 | 0,000 | -1,469 | 0,049 | 0,000 | -1,147 | 0,018 | 0,000 | 30160 |
| April | 2,095 | 0,042 | 0,000 | -2,690 | 0,051 | 0,000 | -1,303 | 0,018 | 0,000 | 28199 |
| May | 1,741 | 0,043 | 0,000 | -3,382 | 0,052 | 0,000 | -1,709 | 0,019 | 0,000 | 28174 |
| June | 1,173 | 0,043 | 0,000 | -3,723 | 0,051 | 0,000 | -2,208 | 0,019 | 0,000 | 28632 |
| July | 0,549 | 0,045 | 0,000 | -3,868 | 0,054 | 0,000 | -2,485 | 0,019 | 0,000 | 20275 |
| August | 0,168 | 0,047 | 0,000 | -2,898 | 0,058 | 0,000 | -1,856 | 0,020 | 0,000 | 14742 |
| September | -0,061 | 0,040 | 0,125 | -2,095 | 0,049 | 0,000 | -1,061 | 0,017 | 0,000 | 28055 |
| October | -0,267 | 0,036 | 0,000 | -1,102 | 0,046 | 0,000 | -0,393 | 0,015 | 0,000 | 28989 |
| November | -0,379 | 0,030 | 0,000 | -0,315 | 0,039 | 0,000 | -0,018 | 0,013 | 0,150 | 28393 |
| December | Ref | | | Ref | | | Ref | | | 27097 |
| **Year of TD** | | | | | | | | | | |
| 2008 | -0,755 | 0,080 | 0,000 | -0,249 | 0,081 | 0,002 | -0,153 | 0,037 | 0,000 | 19482 |
| 2009 | -0,671 | 0,062 | 0,000 | -0,321 | 0,062 | 0,000 | -0,184 | 0,029 | 0,000 | 47734 |
| 2010 | -0,434 | 0,059 | 0,000 | 0,249 | 0,061 | 0,000 | 0,324 | 0,027 | 0,000 | 46479 |
| 2011 | 0,189 | 0,053 | 0,000 | -0,151 | 0,057 | 0,008 | -0,077 | 0,024 | 0,001 | 45157 |
| 2012 | Ref | | | Ref | | | Ref | | | 42979 |
| 2013 | -0,284 | 0,054 | 0,000 | -0,138 | 0,059 | 0,018 | -0,056 | 0,024 | 0,022 | 41683 |
| 2014 | 0,485 | 0,061 | 0,000 | -0,763 | 0,062 | 0,000 | -0,342 | 0,028 | 0,000 | 41969 |
| 2015 | 0,915 | 0,069 | 0,000 | -0,638 | 0,068 | 0,000 | -0,358 | 0,032 | 0,000 | 34541 |
| **Geographic area** | | | | | | | | | | |
| Intensive areas in western France | Ref | | | Ref | | | Ref | | | 156114 |
| Field crops areas | -0,017 | 0,406 | 0,966 | -1,206 | 0,225 | 0,000 | -0,446 | 0,096 | 0,000 | 18489 |
| Grassland areas in northern Massif Central | -0,805 | 0,459 | 0,080 | -0,666 | 0,257 | 0,010 | -0,569 | 0,110 | 0,000 | 12256 |
| Grassland areas in northwestern France | -1,101 | 0,269 | 0,000 | -0,619 | 0,150 | 0,000 | -0,282 | 0,064 | 0,000 | 48111 |
| Intensive piedmont areas | -1,565 | 0,456 | 0,001 | -0,456 | 0,253 | 0,071 | -0,381 | 0,107 | 0,000 | 15777 |
| Mountains wetlands in Massif Central | -2,060 | 0,605 | 0,001 | -0,628 | 0,331 | 0,058 | -0,309 | 0,140 | 0,027 | 9085 |
| Mixed crop-livestock systems in Parisian Basin | -0,238 | 0,242 | 0,326 | -1,066 | 0,135 | 0,000 | -0,450 | 0,058 | 0,000 | 18489 |
| ^1^ *P-*value for comparison with TD in the reference category | | | | | | | | | | |

**Supplementary Table S6.** Model results for the association between extruded linseed exposure status and milk yield (kg/day), milk protein and fat content (g/kg) according to the cow parity***.*** ^1^ Exposure status was gathered 5 categories according to the average daily intake of EL per cow per day during the interval between two TD or between calving and first TD. ^2^ *P*-value for comparison with unexposed population to EL.

|  | Parity | | | | | | | | | | | | | | |
| --- | --- | --- | --- | --- | --- | --- | --- | --- | --- | --- | --- | --- | --- | --- | --- |
|  | 1 (n = 1 373 175) | | | 2 (n = 1 060 457) | | | 3 (n = 695 611) | | | 4 (n = 398 052) | | | 5+ (n = 320 024) | | |
| EL exposure status^1^ | Estimate | SE | *P^2^* | Estimate | SE | *P* | Estimate | SE | *P* | Estimate | SE | *P* | Estimate | SE | *P* |
| Milk Yield (kg/day) | | | | | | | | | | | | | | | |
| Unexposed | ref |  |  | ref |  |  | ref |  |  | ref |  |  | ref |  |  |
| 1-50 | 0.09 | 0.028 | 0.001 | 0.10 | 0.038 | 0.006 | 0.18 | 0.049 | <0.001 | 0.10 | 0.066 | NS | 0.07 | 0.072 | NS |
| 51-300 | 0.40 | 0.017 | <0.001 | 0.54 | 0.024 | <0.001 | 0.59 | 0.031 | <0.001 | 0.61 | 0.042 | <0.001 | 0.53 | 0.046 | <0.001 |
| 301-600 | 0.63 | 0.019 | <0.001 | 0.79 | 0.026 | <0.001 | 0.85 | 0.034 | <0.001 | 0.90 | 0.046 | <0.001 | 0.75 | 0.051 | <0.001 |
| 601-1 500 | 0.82 | 0.024 | <0.001 | 0.99 | 0.033 | <0.001 | 1.05 | 0.043 | <0.001 | 1.09 | 0.059 | <0.001 | 0.89 | 0.065 | <0.001 |
| Milk Fat Content (g/kg) | | | | | | | | | | | | | | | |
| Unexposed | ref |  |  | ref |  |  | ref |  |  | ref |  |  | ref |  |  |
| 1-50 | -0.05 | 0.037 | NS | -0.04 | 0.043 | NS | -0.09 | 0.054 | NS | -0.19 | 0.072 | 0.008 | -0.13 | 0.079 | NS |
| 51-300 | -0.18 | 0.021 | <0.001 | -0.39 | 0.025 | <0.001 | -0.38 | 0.032 | <0.001 | -0.46 | 0.042 | <0.001 | -0.34 | 0.048 | <0.001 |
| 301-600 | -0.30 | 0.023 | <0.001 | -0.55 | 0.028 | <0.001 | -0.60 | 0.036 | <0.001 | -0.75 | 0.048 | <0.001 | -0.58 | 0.054 | <0.001 |
| 601-1 500 | -0.43 | 0.030 | <0.001 | -0.73 | 0.037 | <0.001 | -0.73 | 0.048 | <0.001 | -0.90 | 0.065 | <0.001 | -0.80 | 0.072 | <0.001 |
| Milk Protein Content (g/kg) | | | | | | | | | | | | | | | |
| Unexposed | ref |  |  | ref |  |  | ref |  |  | ref |  |  | ref |  |  |
| 1-50 | -0.01 | 0.015 | NS | 0.00 | 0.018 | NS | -0.02 | 0.022 | NS | -0.07 | 0.028 | 0.009 | 0.01 | 0.031 | NS |
| 51-300 | 0.01 | 0.009 | NS | -0.05 | 0.011 | <0.001 | -0.05 | 0.014 | <0.001 | -0.10 | 0.018 | <0.001 | -0.08 | 0.020 | <0.001 |
| 301-600 | -0.01 | 0.010 | NS | -0.09 | 0.012 | <0.001 | -0.09 | 0.015 | 0.001 | -0.13 | 0.020 | <0.001 | -0.12 | 0.022 | <0.001 |
| 601-1 500 | -0.03 | 0.012 | 0.003 | -0.10 | 0.015 | <0.001 | -0.14 | 0.019 | <0.001 | -0.16 | 0.025 | <0.001 | -0.15 | 0.028 | <0.001 |
|  |  |  |  |  |  |  |  |  |  |  |  |  |  |  |  |
